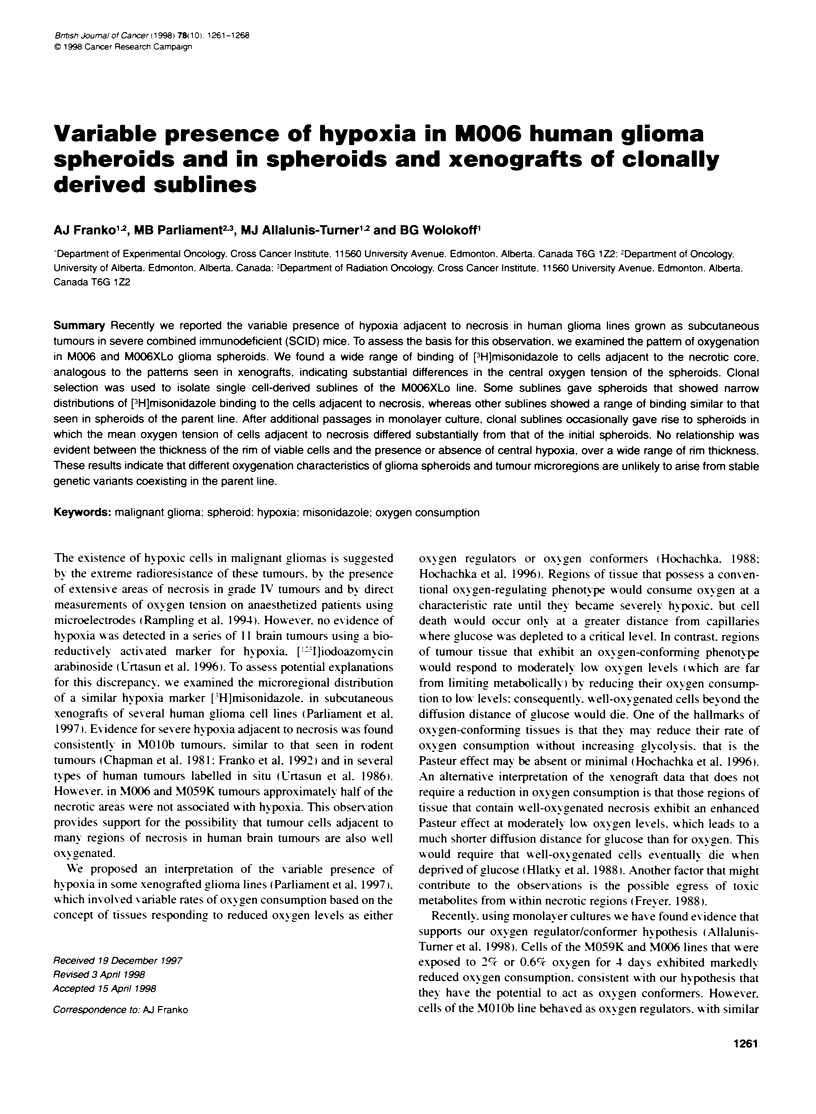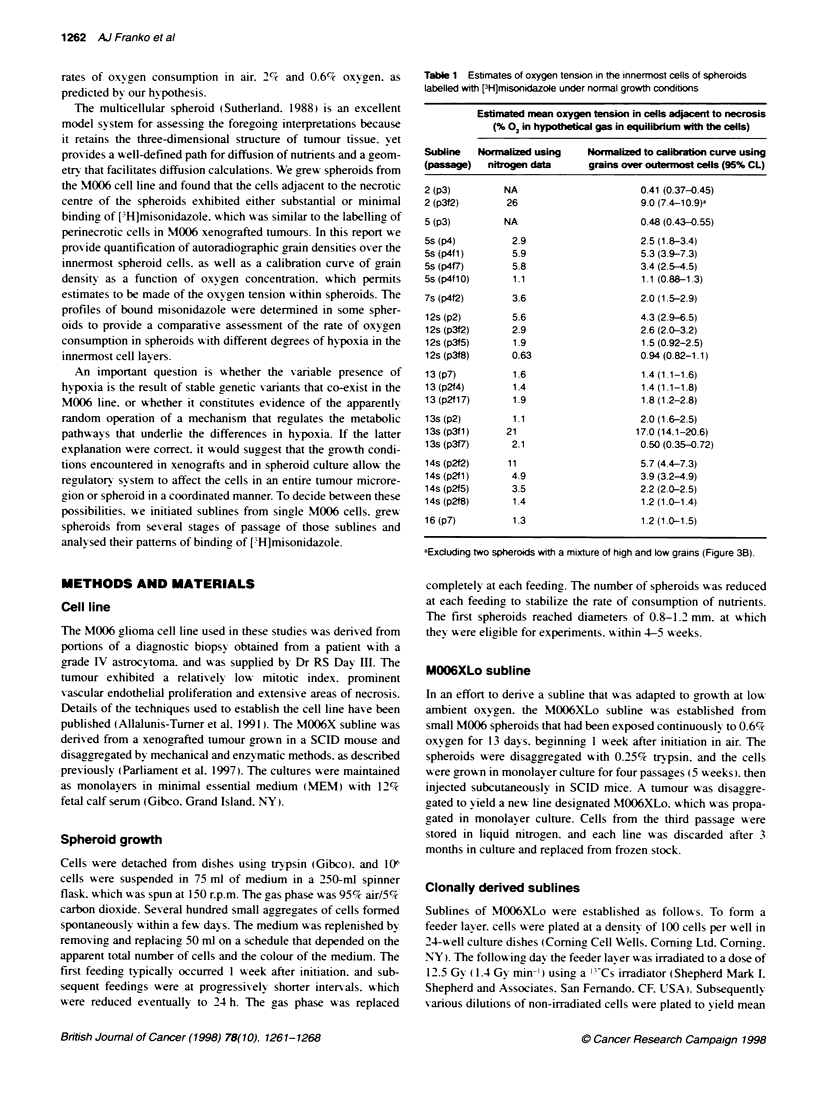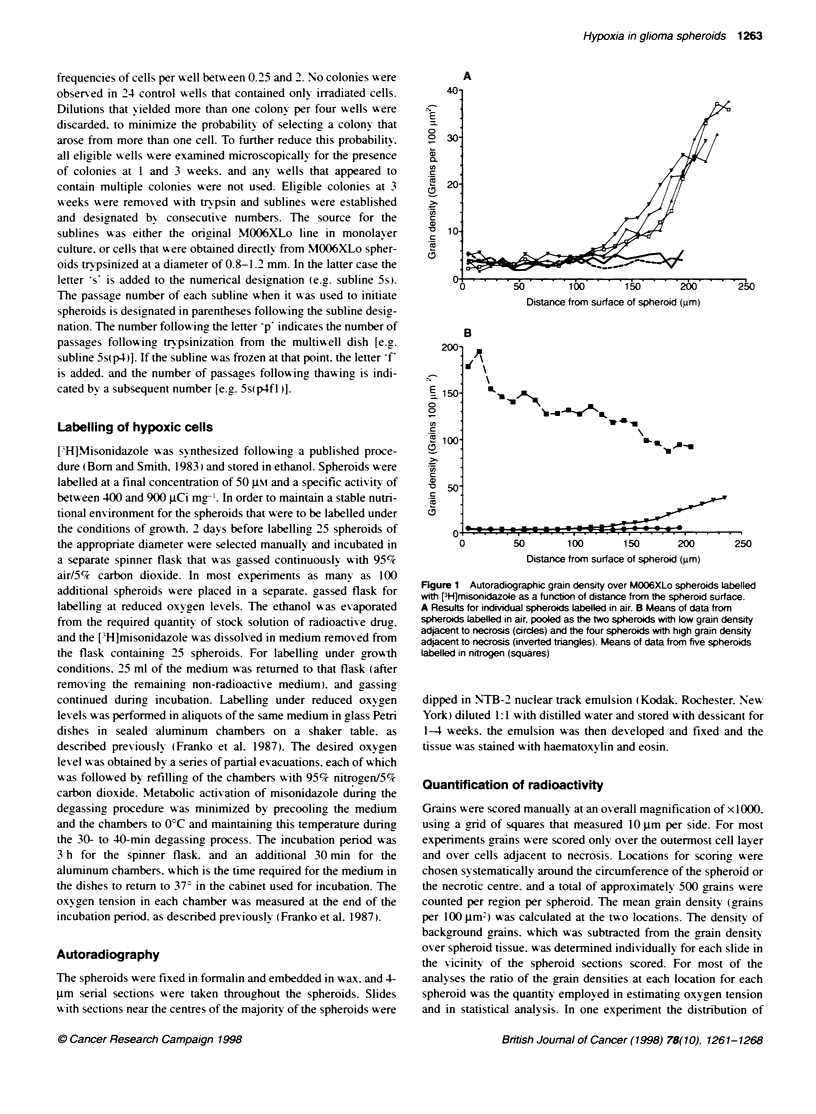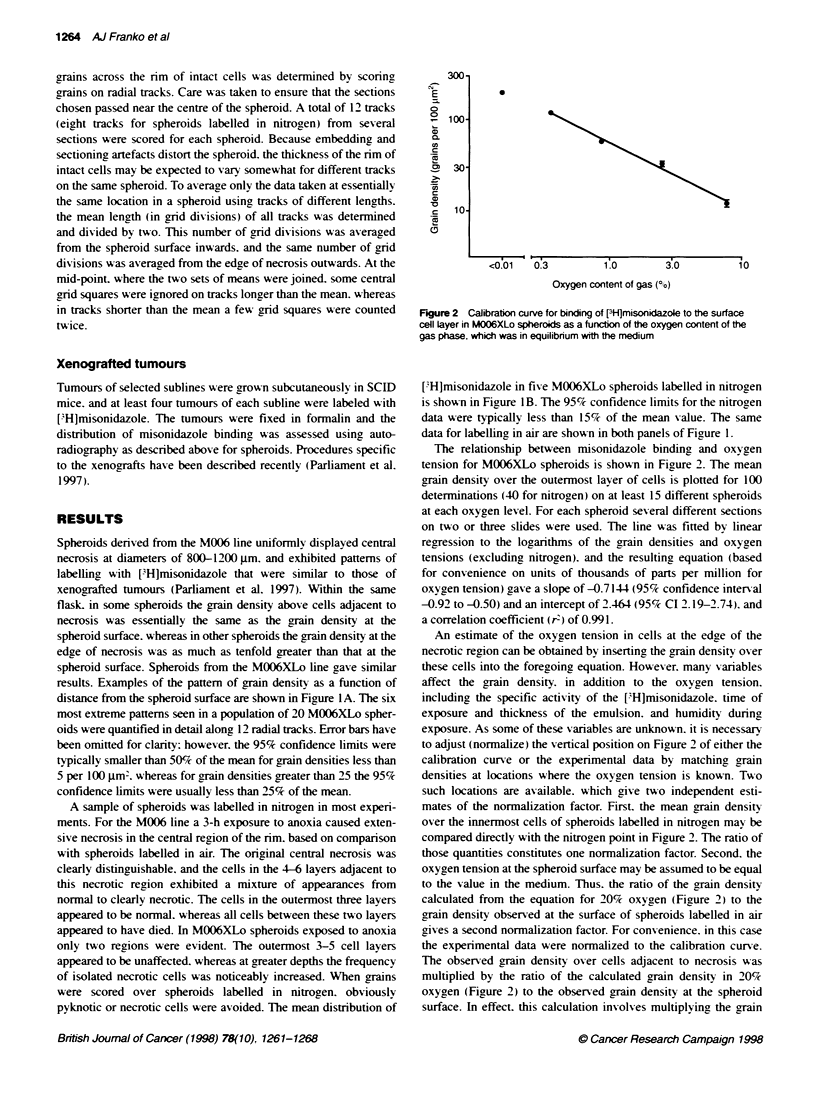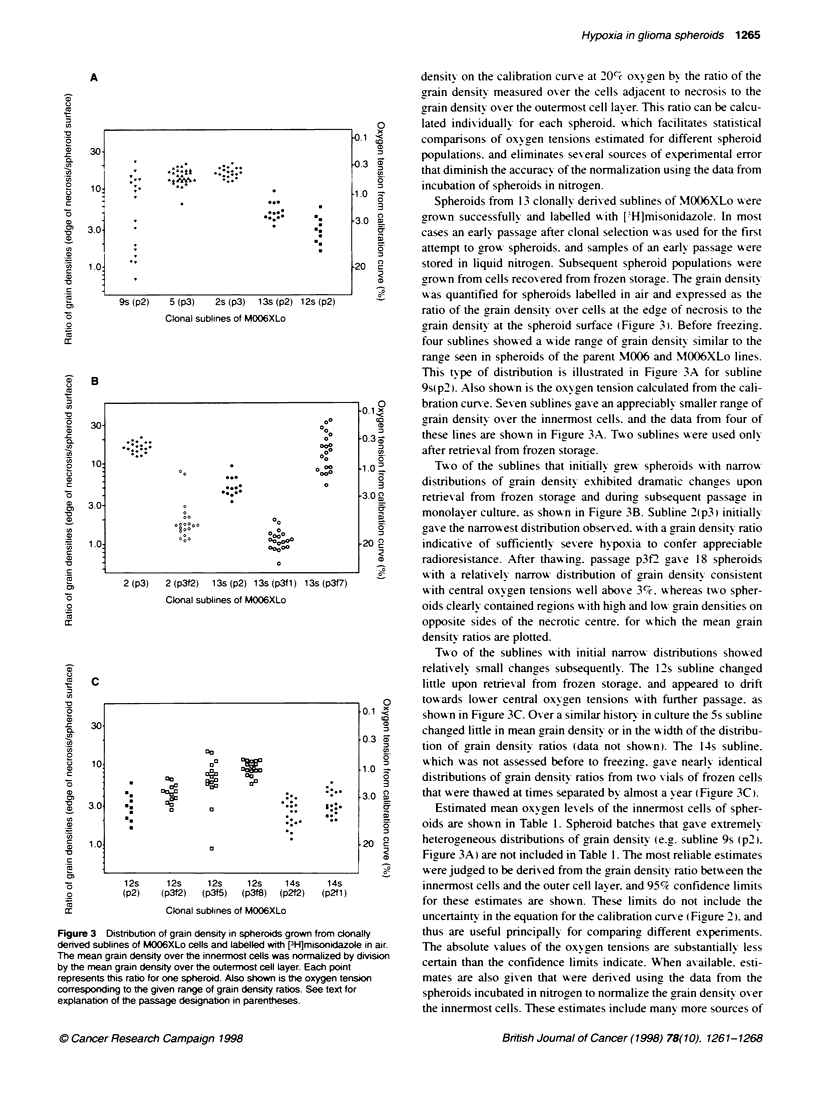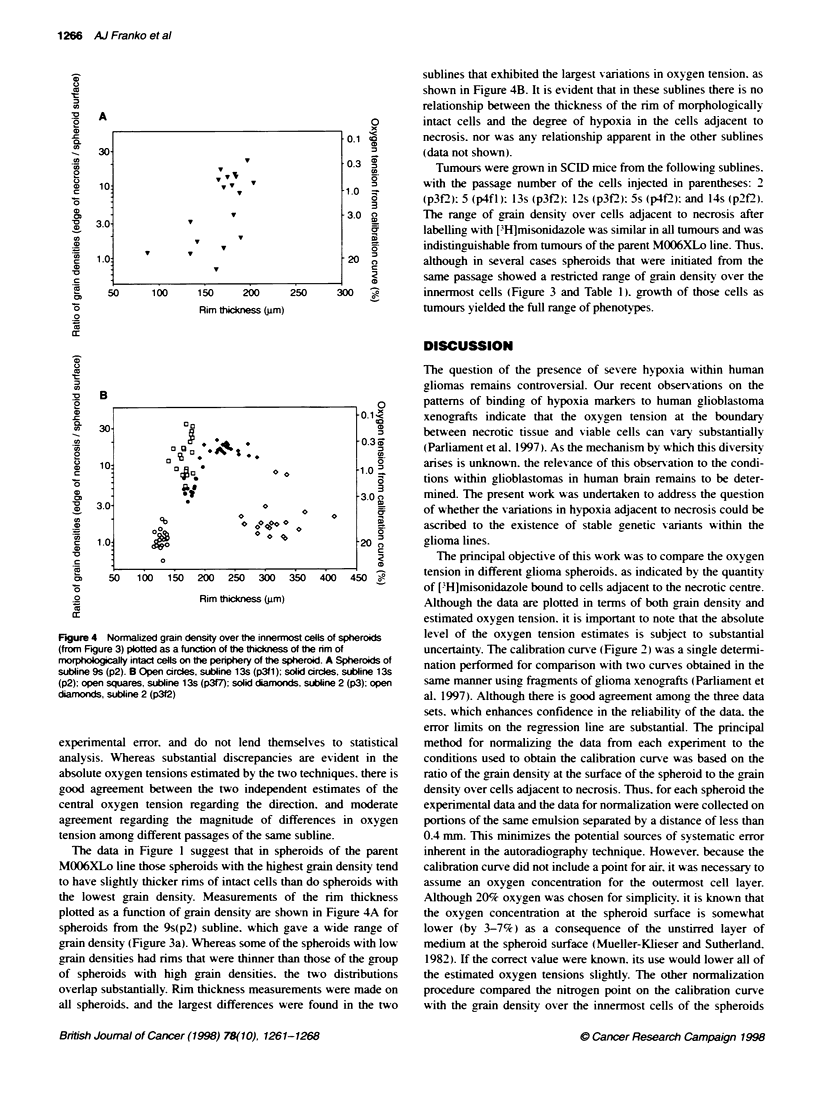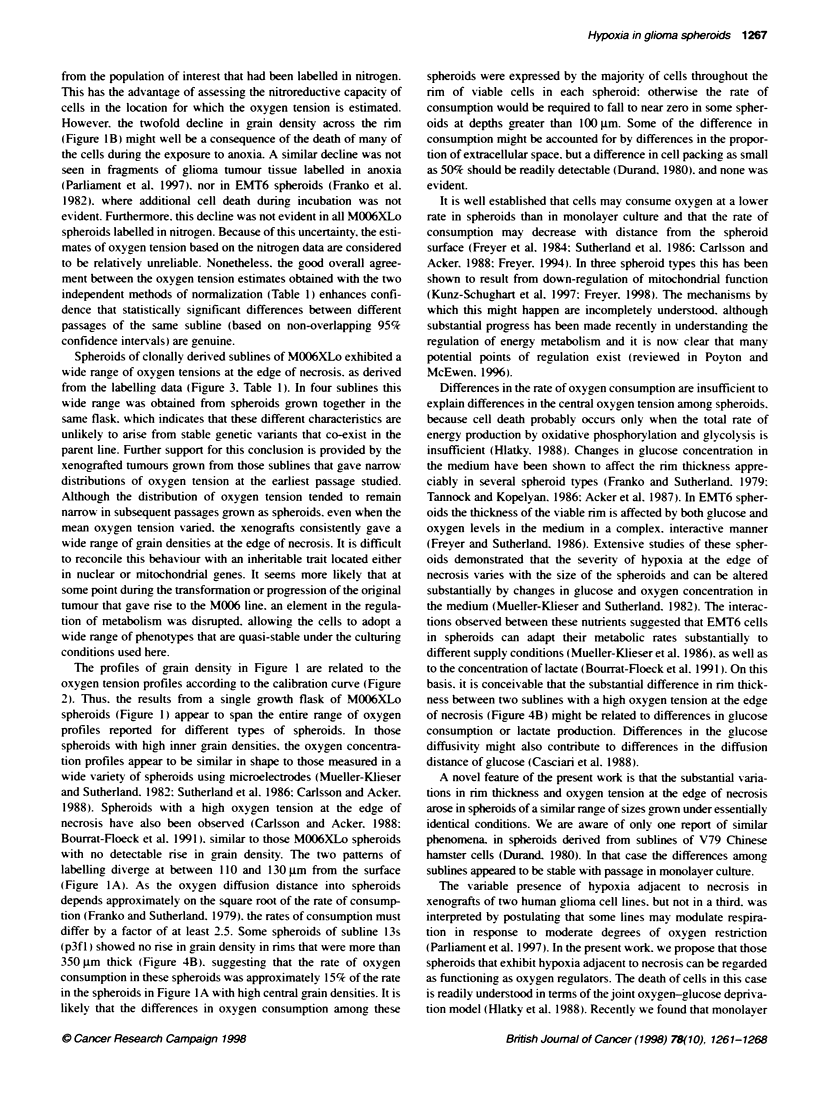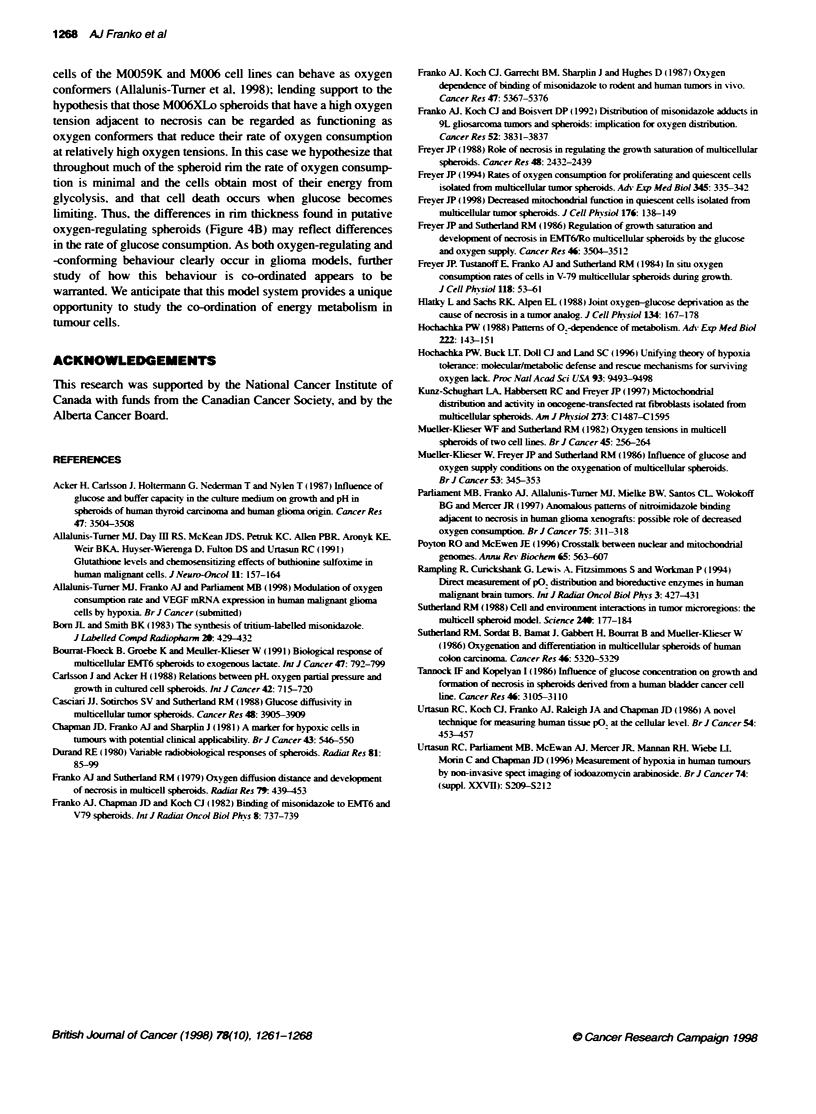# Variable presence of hypoxia in M006 human glioma spheroids and in spheroids and xenografts of clonally derived sublines.

**DOI:** 10.1038/bjc.1998.669

**Published:** 1998-11

**Authors:** A. J. Franko, M. B. Parliament, M. J. Allalunis-Turner, B. G. Wolokoff

**Affiliations:** Department of Experimental Oncology, Cross Cancer Institute, Edmonton, Alberta, Canada.

## Abstract

Recently we reported the variable presence of hypoxia adjacent to necrosis in human glioma lines grown as subcutaneous tumours in severe combined immunodeficient (SCID) mice. To assess the basis for this observation, we examined the pattern of oxygenation in M006 and M006XLo glioma spheroids. We found a wide range of binding of [3H]misonidazole to cells adjacent to the necrotic core, analogous to the patterns seen in xenografts, indicating substantial differences in the central oxygen tension of the spheroids. Clonal selection was used to isolate single cell-derived sublines of the M006XLo line. Some sublines gave spheroids that showed narrow distributions of [3H]misonidazole binding to the cells adjacent to necrosis, whereas other sublines showed a range of binding similar to that seen in spheroids of the parent line. After additional passages in monolayer culture, clonal sublines occasionally gave rise to spheroids in which the mean oxygen tension of cells adjacent to necrosis differed substantially from that of the initial spheroids. No relationship was evident between the thickness of the rim of viable cells and the presence or absence of central hypoxia, over a wide range of rim thickness. These results indicate that different oxygenation characteristics of glioma spheroids and tumour microregions are unlikely to arise from stable genetic variants coexisting in the parent line.


British Journal of Cancer (1998) 78(10), 1261–1268
© 1998 Cancer Research Campaign

### Variable presence of hypoxia in M006 human glioma spheroids and in spheroids and xenografts of clonally derived sublines

**AJ Franko[1,2], MB Parliament[2,3], MJ Allalunis-Turner[1,2] and BG Wolokoff[1]**

[1]Department of Experimental Oncology, Cross Cancer Institute, 11560 University Avenue, Edmonton, Alberta, Canada T6G 1Z2; [2]Department of Oncology, University of Alberta, Edmonton, Alberta, Canada; [3]Department of Radiation Oncology, Cross Cancer Institute, 11560 University Avenue, Edmonton, Alberta, Canada T6G 1Z2

**Summary** Recently we reported the variable presence of hypoxia adjacent to necrosis in human glioma lines grown as subcutaneous tumours in severe combined immunodeficient (SCID) mice. To assess the basis for this observation, we examined the pattern of oxygenation in M006 and M006XLo glioma spheroids. We found a wide range of binding of [$^3$H]misonidazole to cells adjacent to the necrotic core, analogous to the patterns seen in xenografts, indicating substantial differences in the central oxygen tension of the spheroids. Clonal selection was used to isolate single cell-derived sublines of the M006XLo line. Some sublines gave spheroids that showed narrow distributions of [$^3$H]misonidazole binding to the cells adjacent to necrosis, whereas other sublines showed a range of binding similar to that seen in spheroids of the parent line. After additional passages in monolayer culture, clonal sublines occasionally gave rise to spheroids in which the mean oxygen tension of cells adjacent to necrosis differed substantially from that of the initial spheroids. No relationship was evident between the thickness of the rim of viable cells and the presence or absence of central hypoxia, over a wide range of rim thickness. These results indicate that different oxygenation characteristics of glioma spheroids and tumour microregions are unlikely to arise from stable genetic variants coexisting in the parent line.

**Keywords:** malignant glioma; spheroid; hypoxia; misonidazole; oxygen consumption

The existence of hypoxic cells in malignant gliomas is suggested by the extreme radioresistance of these tumours, by the presence of extensive areas of necrosis in grade IV tumours and by direct measurements of oxygen tension on anaesthetized patients using microelectrodes (Rampling et al, 1994). However, no evidence of hypoxia was detected in a series of 11 brain tumours using a bioreductively activated marker for hypoxia, [$^{123}$I]iodoazomycin arabinoside (Urtasun et al, 1996). To assess potential explanations for this discrepancy, we examined the microregional distribution of a similar hypoxia marker [$^3$H]misonidazole, in subcutaneous xenografts of several human glioma cell lines (Parliament et al, 1997). Evidence for severe hypoxia adjacent to necrosis was found consistently in M010b tumours, similar to that seen in rodent tumours (Chapman et al, 1981; Franko et al, 1992) and in several types of human tumours labelled in situ (Urtasun et al, 1986). However, in M006 and M059K tumours approximately half of the necrotic areas were not associated with hypoxia. This observation provides support for the possibility that tumour cells adjacent to many regions of necrosis in human brain tumours are also well oxygenated.

We proposed an interpretation of the variable presence of hypoxia in some xenografted glioma lines (Parliament et al, 1997), which involved variable rates of oxygen consumption based on the concept of tissues responding to reduced oxygen levels as either oxygen regulators or oxygen conformers (Hochachka, 1988; Hochachka et al, 1996). Regions of tissue that possess a conventional oxygen-regulating phenotype would consume oxygen at a characteristic rate until they became severely hypoxic, but cell death would occur only at a greater distance from capillaries where glucose was depleted to a critical level. In contrast, regions of tumour tissue that exhibit an oxygen-conforming phenotype would respond to moderately low oxygen levels (which are far from limiting metabolically) by reducing their oxygen consumption to low levels; consequently, well-oxygenated cells beyond the diffusion distance of glucose would die. One of the hallmarks of oxygen-conforming tissues is that they may reduce their rate of oxygen consumption without increasing glycolysis, that is the Pasteur effect may be absent or minimal (Hochachka et al, 1996). An alternative interpretation of the xenograft data that does not require a reduction in oxygen consumption is that those regions of tissue that contain well-oxygenated necrosis exhibit an enhanced Pasteur effect at moderately low oxygen levels, which leads to a much shorter diffusion distance for glucose than for oxygen. This would require that well-oxygenated cells eventually die when deprived of glucose (Hlatky et al, 1988). Another factor that might contribute to the observations is the possible egress of toxic metabolites from within necrotic regions (Freyer, 1988).

Recently, using monolayer cultures we have found evidence that supports our oxygen regulator/conformer hypothesis (Allalunis-Turner et al, 1998). Cells of the M059K and M006 lines that were exposed to 2% or 0.6% oxygen for 4 days exhibited markedly reduced oxygen consumption, consistent with our hypothesis that they have the potential to act as oxygen conformers. However, cells of the M010b line behaved as oxygen regulators, with similar

*Received 19 December 1997*
*Revised 3 April 1998*
*Accepted 15 April 1998*

*Correspondence to:* AJ Franko rates of oxygen consumption in air, 2% and 0.6% oxygen, as predicted by our hypothesis.

The multicellular spheroid (Sutherland, 1988) is an excellent model system for assessing the foregoing interpretations because it retains the three-dimensional structure of tumour tissue, yet provides a well-defined path for diffusion of nutrients and a geometry that facilitates diffusion calculations. We grew spheroids from the M006 cell line and found that the cells adjacent to the necrotic centre of the spheroids exhibited either substantial or minimal binding of [$^3$H]misonidazole, which was similar to the labelling of perinecrotic cells in M006 xenografted tumours. In this report we provide quantification of autoradiographic grain densities over the innermost spheroid cells, as well as a calibration curve of grain density as a function of oxygen concentration, which permits estimates to be made of the oxygen tension within spheroids. The profiles of bound misonidazole were determined in some spheroids to provide a comparative assessment of the rate of oxygen consumption in spheroids with different degrees of hypoxia in the innermost cell layers.

An important question is whether the variable presence of hypoxia is the result of stable genetic variants that co-exist in the M006 line, or whether it constitutes evidence of the apparently random operation of a mechanism that regulates the metabolic pathways that underlie the differences in hypoxia. If the latter explanation were correct, it would suggest that the growth conditions encountered in xenografts and in spheroid culture allow the regulatory system to affect the cells in an entire tumour microregion or spheroid in a coordinated manner. To decide between these possibilities, we initiated sublines from single M006 cells, grew spheroids from several stages of passage of those sublines and analysed their patterns of binding of [$^3$H]misonidazole.

**Table 1** Estimates of oxygen tension in the innermost cells of spheroids labelled with [$^3$H]misonidazole under normal growth conditions

| | Estimated mean oxygen tension in cells adjacent to necrosis (% $O_2$ in hypothetical gas in equilibrium with the cells) | |
|---|---|---|
| **Subline (passage)** | **Normalized using nitrogen data** | **Normalized to calibration curve using grains over outermost cells (95% CL)** |
| 2 (p3) | NA | 0.41 (0.37–0.45) |
| 2 (p3f2) | 26 | 9.0 (7.4–10.9)[a] |
| 5 (p3) | NA | 0.48 (0.43–0.55) |
| 5s (p4) | 2.9 | 2.5 (1.8–3.4) |
| 5s (p4f1) | 5.9 | 5.3 (3.9–7.3) |
| 5s (p4f7) | 5.8 | 3.4 (2.5–4.5) |
| 5s (p4f10) | 1.1 | 1.1 (0.88–1.3) |
| 7s (p4f2) | 3.6 | 2.0 (1.5–2.9) |
| 12s (p2) | 5.6 | 4.3 (2.9–6.5) |
| 12s (p3f2) | 2.9 | 2.6 (2.0–3.2) |
| 12s (p3f5) | 1.9 | 1.5 (0.92–2.5) |
| 12s (p3f8) | 0.63 | 0.94 (0.82–1.1) |
| 13 (p7) | 1.6 | 1.4 (1.1–1.6) |
| 13 (p2f4) | 1.4 | 1.4 (1.1–1.8) |
| 13 (p2f17) | 1.9 | 1.8 (1.2–2.8) |
| 13s (p2) | 1.1 | 2.0 (1.6–2.5) |
| 13s (p3f1) | 21 | 17.0 (14.1–20.6) |
| 13s (p3f7) | 2.1 | 0.50 (0.35–0.72) |
| 14s (p2f2) | 11 | 5.7 (4.4–7.3) |
| 14s (p2f1) | 4.9 | 3.9 (3.2–4.9) |
| 14s (p2f5) | 3.5 | 2.2 (2.0–2.5) |
| 14s (p2f8) | 1.4 | 1.2 (1.0–1.4) |
| 16 (p7) | 1.3 | 1.2 (1.0–1.5) |

[a]Excluding two spheroids with a mixture of high and low grains (Figure 3B).

### METHODS AND MATERIALS

### Cell line

The M006 glioma cell line used in these studies was derived from portions of a diagnostic biopsy obtained from a patient with a grade IV astrocytoma, and was supplied by Dr RS Day III. The tumour exhibited a relatively low mitotic index, prominent vascular endothelial proliferation and extensive areas of necrosis. Details of the techniques used to establish the cell line have been published (Allalunis-Turner et al, 1991). The M006X subline was derived from a xenografted tumour grown in a SCID mouse and disaggregated by mechanical and enzymatic methods, as described previously (Parliament et al, 1997). The cultures were maintained as monolayers in minimal essential medium (MEM) with 12% fetal calf serum (Gibco, Grand Island, NY).

### Spheroid growth

Cells were detached from dishes using trypsin (Gibco), and $10^6$ cells were suspended in 75 ml of medium in a 250-ml spinner flask, which was spun at 150 r.p.m. The gas phase was 95% air/5% carbon dioxide. Several hundred small aggregates of cells formed spontaneously within a few days. The medium was replenished by removing and replacing 50 ml on a schedule that depended on the apparent total number of cells and the colour of the medium. The first feeding typically occurred 1 week after initiation, and subsequent feedings were at progressively shorter intervals, which were reduced eventually to 24 h. The gas phase was replaced completely at each feeding. The number of spheroids was reduced at each feeding to stabilize the rate of consumption of nutrients. The first spheroids reached diameters of 0.8–1.2 mm, at which they were eligible for experiments, within 4–5 weeks.

### M006XLo subline

In an effort to derive a subline that was adapted to growth at low ambient oxygen, the M006XLo subline was established from small M006 spheroids that had been exposed continuously to 0.6% oxygen for 13 days, beginning 1 week after initiation in air. The spheroids were disaggregated with 0.25% trypsin, and the cells were grown in monolayer culture for four passages (5 weeks), then injected subcutaneously in SCID mice. A tumour was disaggregated to yield a new line designated M006XLo, which was propagated in monolayer culture. Cells from the third passage were stored in liquid nitrogen, and each line was discarded after 3 months in culture and replaced from frozen stock.

### Clonally derived sublines

Sublines of M006XLo were established as follows. To form a feeder layer, cells were plated at a density of 100 cells per well in 24-well culture dishes (Corning Cell Wells, Corning Ltd, Corning, NY). The following day the feeder layer was irradiated to a dose of 12.5 Gy (1.4 Gy min$^{-1}$) using a $^{137}$Cs irradiator (Shepherd Mark I, Shepherd and Associates, San Fernando, CF, USA). Subsequently various dilutions of non-irradiated cells were plated to yield mean

© Cancer Research Campaign 1998

frequencies of cells per well between 0.25 and 2. No colonies were observed in 24 control wells that contained only irradiated cells. Dilutions that yielded more than one colony per four wells were discarded, to minimize the probability of selecting a colony that arose from more than one cell. To further reduce this probability, all eligible wells were examined microscopically for the presence of colonies at 1 and 3 weeks, and any wells that appeared to contain multiple colonies were not used. Eligible colonies at 3 weeks were removed with trypsin and sublines were established and designated by consecutive numbers. The source for the sublines was either the original M006XLo line in monolayer culture, or cells that were obtained directly from M006XLo spheroids trypsinized at a diameter of 0.8–1.2 mm. In the latter case the letter 's' is added to the numerical designation (e.g. subline 5s). The passage number of each subline when it was used to initiate spheroids is designated in parentheses following the subline designation. The number following the letter 'p' indicates the number of passages following trypsinization from the multiwell dish [e.g. subline 5s(p4)]. If the subline was frozen at that point, the letter 'f' is added, and the number of passages following thawing is indicated by a subsequent number [e.g. 5s(p4f1)].

### Labelling of hypoxic cells

[$^3$H]Misonidazole was synthesized following a published procedure (Born and Smith, 1983) and stored in ethanol. Spheroids were labelled at a final concentration of 50 μM and a specific activity of between 400 and 900 μCi mg$^{-1}$. In order to maintain a stable nutritional environment for the spheroids that were to be labelled under the conditions of growth, 2 days before labelling 25 spheroids of the appropriate diameter were selected manually and incubated in a separate spinner flask that was gassed continuously with 95% air/5% carbon dioxide. In most experiments as many as 100 additional spheroids were placed in a separate, gassed flask for labelling at reduced oxygen levels. The ethanol was evaporated from the required quantity of stock solution of radioactive drug, and the [$^3$H]misonidazole was dissolved in medium removed from the flask containing 25 spheroids. For labelling under growth conditions, 25 ml of the medium was returned to that flask (after removing the remaining non-radioactive medium), and gassing continued during incubation. Labelling under reduced oxygen levels was performed in aliquots of the same medium in glass Petri dishes in sealed aluminum chambers on a shaker table, as described previously (Franko et al, 1987). The desired oxygen level was obtained by a series of partial evacuations, each of which was followed by refilling of the chambers with 95% nitrogen/5% carbon dioxide. Metabolic activation of misonidazole during the degassing procedure was minimized by precooling the medium and the chambers to 0°C and maintaining this temperature during the 30- to 40-min degassing process. The incubation period was 3 h for the spinner flask, and an additional 30 min for the aluminum chambers, which is the time required for the medium in the dishes to return to 37° in the cabinet used for incubation. The oxygen tension in each chamber was measured at the end of the incubation period, as described previously (Franko et al, 1987).

### Autoradiography

The spheroids were fixed in formalin and embedded in wax, and 4-μm serial sections were taken throughout the spheroids. Slides with sections near the centres of the majority of the spheroids were

**Figure 1** Autoradiographic grain density over M006XLo spheroids labelled with [$^3$H]misonidazole as a function of distance from the spheroid surface. **A** Results for individual spheroids labelled in air. **B** Means of data from spheroids labelled in air, pooled as the two spheroids with low grain density adjacent to necrosis (circles) and the four spheroids with high grain density adjacent to necrosis (inverted triangles). Means of data from five spheroids labelled in nitrogen (squares)

dipped in NTB-2 nuclear track emulsion (Kodak, Rochester, New York) diluted 1:1 with distilled water and stored with dessicant for 1–4 weeks, the emulsion was then developed and fixed and the tissue was stained with haematoxylin and eosin.

### Quantification of radioactivity

Grains were scored manually at an overall magnification of ×1000, using a grid of squares that measured 10 μm per side. For most experiments grains were scored only over the outermost cell layer and over cells adjacent to necrosis. Locations for scoring were chosen systematically around the circumference of the spheroid or the necrotic centre, and a total of approximately 500 grains were counted per region per spheroid. The mean grain density (grains per 100 μm$^{-2}$) was calculated at the two locations. The density of background grains, which was subtracted from the grain density over spheroid tissue, was determined individually for each slide in the vicinity of the spheroid sections scored. For most of the analyses the ratio of the grain densities at each location for each spheroid was the quantity employed in estimating oxygen tension and in statistical analysis. In one experiment the distribution of

© Cancer Research Campaign 1998 

grains across the rim of intact cells was determined by scoring grains on radial tracks. Care was taken to ensure that the sections chosen passed near the centre of the spheroid. A total of 12 tracks (eight tracks for spheroids labelled in nitrogen) from several sections were scored for each spheroid. Because embedding and sectioning artefacts distort the spheroid. the thickness of the rim of intact cells may be expected to vary somewhat for different tracks on the same spheroid. To average only the data taken at essentially the same location in a spheroid using tracks of different lengths. the mean length (in grid divisions) of all tracks was determined and divided by two. This number of grid divisions was averaged from the spheroid surface inwards. and the same number of grid divisions was averaged from the edge of necrosis outwards. At the mid-point. where the two sets of means were joined. some central grid squares were ignored on tracks longer than the mean. whereas in tracks shorter than the mean a few grid squares were counted twice.

**Figure 2** Calibration curve for binding of [³H]misonidazole to the surface cell layer in M006XLo spheroids as a function of the oxygen content of the gas phase, which was in equilibrium with the medium

### Xenografted tumours

Tumours of selected sublines were grown subcutaneously in SCID mice. and at least four tumours of each subline were labeled with [³H]misonidazole. The tumours were fixed in formalin and the distribution of misonidazole binding was assessed using autoradiography as described above for spheroids. Procedures specific to the xenografts have been described recently (Parliament et al. 1997).

### RESULTS

Spheroids derived from the M006 line uniformly displayed central necrosis at diameters of 800–1200 µm. and exhibited patterns of labelling with [³H]misonidazole that were similar to those of xenografted tumours (Parliament et al. 1997). Within the same flask. in some spheroids the grain density above cells adjacent to necrosis was essentially the same as the grain density at the spheroid surface. whereas in other spheroids the grain density at the edge of necrosis was as much as tenfold greater than that at the spheroid surface. Spheroids from the M006XLo line gave similar results. Examples of the pattern of grain density as a function of distance from the spheroid surface are shown in Figure 1A. The six most extreme patterns seen in a population of 20 M006XLo spheroids were quantified in detail along 12 radial tracks. Error bars have been omitted for clarity: however. the 95% confidence limits were typically smaller than 50% of the mean for grain densities less than 5 per 100 µm$^2$. whereas for grain densities greater than 25 the 95% confidence limits were usually less than 25% of the mean.

A sample of spheroids was labelled in nitrogen in most experiments. For the M006 line a 3-h exposure to anoxia caused extensive necrosis in the central region of the rim. based on comparison with spheroids labelled in air. The original central necrosis was clearly distinguishable. and the cells in the 4–6 layers adjacent to this necrotic region exhibited a mixture of appearances from normal to clearly necrotic. The cells in the outermost three layers appeared to be normal. whereas all cells between these two layers appeared to have died. In M006XLo spheroids exposed to anoxia only two regions were evident. The outermost 3–5 cell layers appeared to be unaffected. whereas at greater depths the frequency of isolated necrotic cells was noticeably increased. When grains were scored over spheroids labelled in nitrogen. obviously pyknotic or necrotic cells were avoided. The mean distribution of [³H]misonidazole in five M006XLo spheroids labelled in nitrogen is shown in Figure 1B. The 95% confidence limits for the nitrogen data were typically less than 15% of the mean value. The same data for labelling in air are shown in both panels of Figure 1.

The relationship between misonidazole binding and oxygen tension for M006XLo spheroids is shown in Figure 2. The mean grain density over the outermost layer of cells is plotted for 100 determinations (40 for nitrogen) on at least 15 different spheroids at each oxygen level. For each spheroid several different sections on two or three slides were used. The line was fitted by linear regression to the logarithms of the grain densities and oxygen tensions (excluding nitrogen). and the resulting equation (based for convenience on units of thousands of parts per million for oxygen tension) gave a slope of –0.7144 (95% confidence interval –0.92 to –0.50) and an intercept of 2.464 (95% CI 2.19–2.74). and a correlation coefficient ($r^2$) of 0.991.

An estimate of the oxygen tension in cells at the edge of the necrotic region can be obtained by inserting the grain density over these cells into the foregoing equation. However. many variables affect the grain density. in addition to the oxygen tension. including the specific activity of the [³H]misonidazole. time of exposure and thickness of the emulsion. and humidity during exposure. As some of these variables are unknown. it is necessary to adjust (normalize) the vertical position on Figure 2 of either the calibration curve or the experimental data by matching grain densities at locations where the oxygen tension is known. Two such locations are available. which give two independent estimates of the normalization factor. First. the mean grain density over the innermost cells of spheroids labelled in nitrogen may be compared directly with the nitrogen point in Figure 2. The ratio of those quantities constitutes one normalization factor. Second. the oxygen tension at the spheroid surface may be assumed to be equal to the value in the medium. Thus. the ratio of the grain density calculated from the equation for 20% oxygen (Figure 2) to the grain density observed at the surface of spheroids labelled in air gives a second normalization factor. For convenience. in this case the experimental data were normalized to the calibration curve. The observed grain density over cells adjacent to necrosis was multiplied by the ratio of the calculated grain density in 20% oxygen (Figure 2) to the observed grain density at the spheroid surface. In effect. this calculation involves multiplying the grain

© Cancer Research Campaign 1998

**Figure 3** Distribution of grain density in spheroids grown from clonally derived sublines of M006XLo cells and labelled with [$^3$H]misonidazole in air. The mean grain density over the innermost cells was normalized by division by the mean grain density over the outermost cell layer. Each point represents this ratio for one spheroid. Also shown is the oxygen tension corresponding to the given range of grain density ratios. See text for explanation of the passage designation in parentheses.

density on the calibration curve at 20% oxygen by the ratio of the grain density measured over the cells adjacent to necrosis to the grain density over the outermost cell layer. This ratio can be calculated individually for each spheroid, which facilitates statistical comparisons of oxygen tensions estimated for different spheroid populations, and eliminates several sources of experimental error that diminish the accuracy of the normalization using the data from incubation of spheroids in nitrogen.

Spheroids from 13 clonally derived sublines of M006XLo were grown successfully and labelled with [$^3$H]misonidazole. In most cases an early passage after clonal selection was used for the first attempt to grow spheroids, and samples of an early passage were stored in liquid nitrogen. Subsequent spheroid populations were grown from cells recovered from frozen storage. The grain density was quantified for spheroids labelled in air and expressed as the ratio of the grain density over cells at the edge of necrosis to the grain density at the spheroid surface (Figure 3). Before freezing, four sublines showed a wide range of grain density similar to the range seen in spheroids of the parent M006 and M006XLo lines. This type of distribution is illustrated in Figure 3A for subline 9s(p2). Also shown is the oxygen tension calculated from the calibration curve. Seven sublines gave an appreciably smaller range of grain density over the innermost cells, and the data from four of these lines are shown in Figure 3A. Two sublines were used only after retrieval from frozen storage.

Two of the sublines that initially grew spheroids with narrow distributions of grain density exhibited dramatic changes upon retrieval from frozen storage and during subsequent passage in monolayer culture, as shown in Figure 3B. Subline 2(p3) initially gave the narrowest distribution observed, with a grain density ratio indicative of sufficiently severe hypoxia to confer appreciable radioresistance. After thawing, passage p3f2 gave 18 spheroids with a relatively narrow distribution of grain density consistent with central oxygen tensions well above 3%, whereas two spheroids clearly contained regions with high and low grain densities on opposite sides of the necrotic centre, for which the mean grain density ratios are plotted.

Two of the sublines with initial narrow distributions showed relatively small changes subsequently. The 12s subline changed little upon retrieval from frozen storage, and appeared to drift towards lower central oxygen tensions with further passage, as shown in Figure 3C. Over a similar history in culture the 5s subline changed little in mean grain density or in the width of the distribution of grain density ratios (data not shown). The 14s subline, which was not assessed before to freezing, gave nearly identical distributions of grain density ratios from two vials of frozen cells that were thawed at times separated by almost a year (Figure 3C).

Estimated mean oxygen levels of the innermost cells of spheroids are shown in Table 1. Spheroid batches that gave extremely heterogeneous distributions of grain density (e.g. subline 9s (p2), Figure 3A) are not included in Table 1. The most reliable estimates were judged to be derived from the grain density ratio between the innermost cells and the outer cell layer, and 95% confidence limits for these estimates are shown. These limits do not include the uncertainty in the equation for the calibration curve (Figure 2), and thus are useful principally for comparing different experiments. The absolute values of the oxygen tensions are substantially less certain than the confidence limits indicate. When available, estimates are also given that were derived using the data from the spheroids incubated in nitrogen to normalize the grain density over the innermost cells. These estimates include many more sources of

**Figure 4** Normalized grain density over the innermost cells of spheroids (from Figure 3) plotted as a function of the thickness of the rim of morphologically intact cells on the periphery of the spheroid. **A** Spheroids of subline 9s (p2). **B** Open circles, subline 13s (p3f1); solid circles, subline 13s (p2); open squares, subline 13s (p3f7); solid diamonds, subline 2 (p3); open diamonds, subline 2 (p3f2)

experimental error, and do not lend themselves to statistical analysis. Whereas substantial discrepancies are evident in the absolute oxygen tensions estimated by the two techniques, there is good agreement between the two independent estimates of the central oxygen tension regarding the direction, and moderate agreement regarding the magnitude of differences in oxygen tension among different passages of the same subline.

The data in Figure 1 suggest that in spheroids of the parent M006XLo line those spheroids with the highest grain density tend to have slightly thicker rims of intact cells than do spheroids with the lowest grain density. Measurements of the rim thickness plotted as a function of grain density are shown in Figure 4A for spheroids from the 9s(p2) subline, which gave a wide range of grain density (Figure 3a). Whereas some of the spheroids with low grain densities had rims that were thinner than those of the group of spheroids with high grain densities, the two distributions overlap substantially. Rim thickness measurements were made on all spheroids, and the largest differences were found in the two sublines that exhibited the largest variations in oxygen tension, as shown in Figure 4B. It is evident that in these sublines there is no relationship between the thickness of the rim of morphologically intact cells and the degree of hypoxia in the cells adjacent to necrosis, nor was any relationship apparent in the other sublines (data not shown).

Tumours were grown in SCID mice from the following sublines, with the passage number of the cells injected in parentheses: 2 (p3f2); 5 (p4f1); 13s (p3f2); 12s (p3f2); 5s (p4f2); and 14s (p2f2). The range of grain density over cells adjacent to necrosis after labelling with [$^3$H]misonidazole was similar in all tumours and was indistinguishable from tumours of the parent M006XLo line. Thus, although in several cases spheroids that were initiated from the same passage showed a restricted range of grain density over the innermost cells (Figure 3 and Table 1), growth of those cells as tumours yielded the full range of phenotypes.

### DISCUSSION

The question of the presence of severe hypoxia within human gliomas remains controversial. Our recent observations on the patterns of binding of hypoxia markers to human glioblastoma xenografts indicate that the oxygen tension at the boundary between necrotic tissue and viable cells can vary substantially (Parliament et al, 1997). As the mechanism by which this diversity arises is unknown, the relevance of this observation to the conditions within glioblastomas in human brain remains to be determined. The present work was undertaken to address the question of whether the variations in hypoxia adjacent to necrosis could be ascribed to the existence of stable genetic variants within the glioma lines.

The principal objective of this work was to compare the oxygen tension in different glioma spheroids, as indicated by the quantity of [$^3$H]misonidazole bound to cells adjacent to the necrotic centre. Although the data are plotted in terms of both grain density and estimated oxygen tension, it is important to note that the absolute level of the oxygen tension estimates is subject to substantial uncertainty. The calibration curve (Figure 2) was a single determination performed for comparison with two curves obtained in the same manner using fragments of glioma xenografts (Parliament et al, 1997). Although there is good agreement among the three data sets, which enhances confidence in the reliability of the data, the error limits on the regression line are substantial. The principal method for normalizing the data from each experiment to the conditions used to obtain the calibration curve was based on the ratio of the grain density at the surface of the spheroid to the grain density over cells adjacent to necrosis. Thus, for each spheroid the experimental data and the data for normalization were collected on portions of the same emulsion separated by a distance of less than 0.4 mm. This minimizes the potential sources of systematic error inherent in the autoradiography technique. However, because the calibration curve did not include a point for air, it was necessary to assume an oxygen concentration for the outermost cell layer. Although 20% oxygen was chosen for simplicity, it is known that the oxygen concentration at the spheroid surface is somewhat lower (by 3–7%) as a consequence of the unstirred layer of medium at the spheroid surface (Mueller-Klieser and Sutherland, 1982). If the correct value were known, its use would lower all of the estimated oxygen tensions slightly. The other normalization procedure compared the nitrogen point on the calibration curve with the grain density over the innermost cells of the spheroids

© Cancer Research Campaign 1998

from the population of interest that had been labelled in nitrogen. This has the advantage of assessing the nitroreductive capacity of cells in the location for which the oxygen tension is estimated. However, the twofold decline in grain density across the rim (Figure 1B) might well be a consequence of the death of many of the cells during the exposure to anoxia. A similar decline was not seen in fragments of glioma tumour tissue labelled in anoxia (Parliament et al, 1997), nor in EMT6 spheroids (Franko et al, 1982), where additional cell death during incubation was not evident. Furthermore, this decline was not evident in all M006XLo spheroids labelled in nitrogen. Because of this uncertainty, the estimates of oxygen tension based on the nitrogen data are considered to be relatively unreliable. Nonetheless, the good overall agreement between the oxygen tension estimates obtained with the two independent methods of normalization (Table 1) enhances confidence that statistically significant differences between different passages of the same subline (based on non-overlapping 95% confidence intervals) are genuine.

Spheroids of clonally derived sublines of M006XLo exhibited a wide range of oxygen tensions at the edge of necrosis, as derived from the labelling data (Figure 3, Table 1). In four sublines this wide range was obtained from spheroids grown together in the same flask, which indicates that these different characteristics are unlikely to arise from stable genetic variants that co-exist in the parent line. Further support for this conclusion is provided by the xenografted tumours grown from those sublines that gave narrow distributions of oxygen tension at the earliest passage studied. Although the distribution of oxygen tension tended to remain narrow in subsequent passages grown as spheroids, even when the mean oxygen tension varied, the xenografts consistently gave a wide range of grain densities at the edge of necrosis. It is difficult to reconcile this behaviour with an inheritable trait located either in nuclear or mitochondrial genes. It seems more likely that at some point during the transformation or progression of the original tumour that gave rise to the M006 line, an element in the regulation of metabolism was disrupted, allowing the cells to adopt a wide range of phenotypes that are quasi-stable under the culturing conditions used here.

The profiles of grain density in Figure 1 are related to the oxygen tension profiles according to the calibration curve (Figure 2). Thus, the results from a single growth flask of M006XLo spheroids (Figure 1) appear to span the entire range of oxygen profiles reported for different types of spheroids. In those spheroids with high inner grain densities, the oxygen concentration profiles appear to be similar in shape to those measured in a wide variety of spheroids using microelectrodes (Mueller-Klieser and Sutherland, 1982; Sutherland et al, 1986; Carlsson and Acker, 1988). Spheroids with a high oxygen tension at the edge of necrosis have also been observed (Carlsson and Acker, 1988; Bourrat-Floeck et al, 1991), similar to those M006XLo spheroids with no detectable rise in grain density. The two patterns of labelling diverge at between 110 and 130 µm from the surface (Figure 1A). As the oxygen diffusion distance into spheroids depends approximately on the square root of the rate of consumption (Franko and Sutherland, 1979), the rates of consumption must differ by a factor of at least 2.5. Some spheroids of subline 13s (p3f1) showed no rise in grain density in rims that were more than 350 µm thick (Figure 4B), suggesting that the rate of oxygen consumption in these spheroids was approximately 15% of the rate in the spheroids in Figure 1A with high central grain densities. It is likely that the differences in oxygen consumption among these spheroids were expressed by the majority of cells throughout the rim of viable cells in each spheroid; otherwise the rate of consumption would be required to fall to near zero in some spheroids at depths greater than 100 µm. Some of the difference in consumption might be accounted for by differences in the proportion of extracellular space, but a difference in cell packing as small as 50% should be readily detectable (Durand, 1980), and none was evident.

It is well established that cells may consume oxygen at a lower rate in spheroids than in monolayer culture and that the rate of consumption may decrease with distance from the spheroid surface (Freyer et al, 1984; Sutherland et al, 1986; Carlsson and Acker, 1988; Freyer, 1994). In three spheroid types this has been shown to result from down-regulation of mitochondrial function (Kunz-Schughart et al, 1997; Freyer, 1998). The mechanisms by which this might happen are incompletely understood, although substantial progress has been made recently in understanding the regulation of energy metabolism and it is now clear that many potential points of regulation exist (reviewed in Poyton and McEwen, 1996).

Differences in the rate of oxygen consumption are insufficient to explain differences in the central oxygen tension among spheroids, because cell death probably occurs only when the total rate of energy production by oxidative phosphorylation and glycolysis is insufficient (Hlatky, 1988). Changes in glucose concentration in the medium have been shown to affect the rim thickness appreciably in several spheroid types (Franko and Sutherland, 1979; Tannock and Kopelyan, 1986; Acker et al, 1987). In EMT6 spheroids the thickness of the viable rim is affected by both glucose and oxygen levels in the medium in a complex, interactive manner (Freyer and Sutherland, 1986). Extensive studies of these spheroids demonstrated that the severity of hypoxia at the edge of necrosis varies with the size of the spheroids and can be altered substantially by changes in glucose and oxygen concentration in the medium (Mueller-Klieser and Sutherland, 1982). The interactions observed between these nutrients suggested that EMT6 cells in spheroids can adapt their metabolic rates substantially to different supply conditions (Mueller-Klieser et al, 1986), as well as to the concentration of lactate (Bourrat-Floeck et al, 1991). On this basis, it is conceivable that the substantial difference in rim thickness between two sublines with a high oxygen tension at the edge of necrosis (Figure 4B) might be related to differences in glucose consumption or lactate production. Differences in the glucose diffusivity might also contribute to differences in the diffusion distance of glucose (Casciari et al, 1988).

A novel feature of the present work is that the substantial variations in rim thickness and oxygen tension at the edge of necrosis arose in spheroids of a similar range of sizes grown under essentially identical conditions. We are aware of only one report of similar phenomena, in spheroids derived from sublines of V79 Chinese hamster cells (Durand, 1980). In that case the differences among sublines appeared to be stable with passage in monolayer culture.

The variable presence of hypoxia adjacent to necrosis in xenografts of two human glioma cell lines, but not in a third, was interpreted by postulating that some lines may modulate respiration in response to moderate degrees of oxygen restriction (Parliament et al, 1997). In the present work, we propose that those spheroids that exhibit hypoxia adjacent to necrosis can be regarded as functioning as oxygen regulators. The death of cells in this case is readily understood in terms of the joint oxygen–glucose deprivation model (Hlatky et al, 1988). Recently we found that monolayer

© Cancer Research Campaign 1998

cells of the M0059K and M006 cell lines can behave as oxygen conformers (Allalunis-Turner et al, 1998); lending support to the hypothesis that those M006XLo spheroids that have a high oxygen tension adjacent to necrosis can be regarded as functioning as oxygen conformers that reduce their rate of oxygen consumption at relatively high oxygen tensions. In this case we hypothesize that throughout much of the spheroid rim the rate of oxygen consumption is minimal and the cells obtain most of their energy from glycolysis, and that cell death occurs when glucose becomes limiting. Thus, the differences in rim thickness found in putative oxygen-regulating spheroids (Figure 4B) may reflect differences in the rate of glucose consumption. As both oxygen-regulating and -conforming behaviour clearly occur in glioma models, further study of how this behaviour is co-ordinated appears to be warranted. We anticipate that this model system provides a unique opportunity to study the co-ordination of energy metabolism in tumour cells.

### ACKNOWLEDGEMENTS

This research was supported by the National Cancer Institute of Canada with funds from the Canadian Cancer Society, and by the Alberta Cancer Board.

### REFERENCES

Acker H, Carlsson J, Holtermann G, Nederman T and Nylen T (1987) Influence of glucose and buffer capacity in the culture medium on growth and pH in spheroids of human thyroid carcinoma and human glioma origin. *Cancer Res* **47**: 3504–3508

Allalunis-Turner MJ, Day III RS, McKean JDS, Petruk KC, Allen PBR, Aronyk KE, Weir BKA, Huyser-Wierenga D, Fulton DS and Urtasun RC (1991) Glutathione levels and chemosensitizing effects of buthionine sulfoxime in human malignant cells. *J Neuro-Oncol* **11**: 157–164

Allalunis-Turner MJ, Franko AJ and Parliament MB (1998) Modulation of oxygen consumption rate and VEGF mRNA expression in human malignant glioma cells by hypoxia. *Br J Cancer* (submitted)

Born JL and Smith BK (1983) The synthesis of tritium-labelled misonidazole. *J Labelled Compd Radiopharm* **20**: 429–432

Bourrat-Floeck B, Groebe K and Meuller-Klieser W (1991) Biological response of multicellular EMT6 spheroids to exogenous lactate. *Int J Cancer* **47**: 792–799

Carlsson J and Acker H (1988) Relations between pH, oxygen partial pressure and growth in cultured cell spheroids. *Int J Cancer* **42**: 715–720

Casciari JJ, Sotirchos SV and Sutherland RM (1988) Glucose diffusivity in multicellular tumor spheroids. *Cancer Res* **48**: 3905–3909

Chapman JD, Franko AJ and Sharplin J (1981) A marker for hypoxic cells in tumours with potential clinical applicability. *Br J Cancer* **43**: 546–550

Durand RE (1980) Variable radiobiological responses of spheroids. *Radiat Res* **81**: 85–99

Franko AJ and Sutherland RM (1979) Oxygen diffusion distance and development of necrosis in multicell spheroids. *Radiat Res* **79**: 439–453

Franko AJ, Chapman JD and Koch CJ (1982) Binding of misonidazole to EMT6 and V79 spheroids. *Int J Radiat Oncol Biol Phys* **8**: 737–739

Franko AJ, Koch CJ, Garrecht BM, Sharplin J and Hughes D (1987) Oxygen dependence of binding of misonidazole to rodent and human tumors in vivo. *Cancer Res* **47**: 5367–5376

Franko AJ, Koch CJ and Boisvert DP (1992) Distribution of misonidazole adducts in 9L gliosarcoma tumors and spheroids: implication for oxygen distribution. *Cancer Res* **52**: 3831–3837

Freyer JP (1988) Role of necrosis in regulating the growth saturation of multicellular spheroids. *Cancer Res* **48**: 2432–2439

Freyer JP (1994) Rates of oxygen consumption for proliferating and quiescent cells isolated from multicellular tumor spheroids. *Adv Exp Med Biol* **345**: 335–342

Freyer JP (1998) Decreased mitochondrial function in quiescent cells isolated from multicellular tumor spheroids. *J Cell Physiol* **176**: 138–149

Freyer JP and Sutherland RM (1986) Regulation of growth saturation and development of necrosis in EMT6/Ro multicellular spheroids by the glucose and oxygen supply. *Cancer Res* **46**: 3504–3512

Freyer JP, Tustanoff E, Franko AJ and Sutherland RM (1984) In situ oxygen consumption rates of cells in V-79 multicellular spheroids during growth. *J Cell Physiol* **118**: 53–61

Hlatky L and Sachs RK, Alpen EL (1988) Joint oxygen–glucose deprivation as the cause of necrosis in a tumor analog. *J Cell Physiol* **134**: 167–178

Hochachka PW (1988) Patterns of $O_2$-dependence of metabolism. *Adv Exp Med Biol* **222**: 143–151

Hochachka PW, Buck LT, Doll CJ and Land SC (1996) Unifying theory of hypoxia tolerance: molecular/metabolic defense and rescue mechanisms for surviving oxygen lack. *Proc Natl Acad Sci USA* **93**: 9493–9498

Kunz-Schughart LA, Habbersett RC and Freyer JP (1997) Mictochondrial distribution and activity in oncogene-transfected rat fibroblasts isolated from multicellular spheroids. *Am J Physiol* **273**: C1487–C1595

Mueller-Klieser WF and Sutherland RM (1982) Oxygen tensions in multicell spheroids of two cell lines. *Br J Cancer* **45**: 256–264

Mueller-Klieser W, Freyer JP and Sutherland RM (1986) Influence of glucose and oxygen supply conditions on the oxygenation of multicellular spheroids. *Br J Cancer* **53**: 345–353

Parliament MB, Franko AJ, Allalunis-Turner MJ, Mielke BW, Santos CL, Wolokoff BG and Mercer JR (1997) Anomalous patterns of nitroimidazole binding adjacent to necrosis in human glioma xenografts: possible role of decreased oxygen consumption. *Br J Cancer* **75**: 311–318

Poyton RO and McEwen JE (1996) Crosstalk between nuclear and mitochondrial genomes. *Annu Rev Biochem* **65**: 563–607

Rampling R, Curickshank G, Lewis A, Fitzsimmons S and Workman P (1994) Direct measurement of $pO_2$ distribution and bioreductive enzymes in human malignant brain tumors. *Int J Radiat Oncol Biol Phys* **3**: 427–431

Sutherland RM (1988) Cell and environment interactions in tumor microregions: the multicell spheroid model. *Science* **240**: 177–184

Sutherland RM, Sordat B, Bamat J, Gabbert H, Bourrat B and Mueller-Klieser W (1986) Oxygenation and differentiation in multicellular spheroids of human colon carcinoma. *Cancer Res* **46**: 5320–5329

Tannock IF and Kopelyan I (1986) Influence of glucose concentration on growth and formation of necrosis in spheroids derived from a human bladder cancer cell line. *Cancer Res* **46**: 3105–3110

Urtasun RC, Koch CJ, Franko AJ, Raleigh JA and Chapman JD (1986) A novel technique for measuring human tissue $pO_2$ at the cellular level. *Br J Cancer* **54**: 453–457

Urtasun RC, Parliament MB, McEwan AJ, Mercer JR, Mannan RH, Wiebe LI, Morin C and Chapman JD (1996) Measurement of hypoxia in human tumours by non-invasive spect imaging of iodoazomycin arabinoside. *Br J Cancer* **74**: (suppl. XXVII): S209–S212

© Cancer Research Campaign 1998